# Outcomes after sofosbuvir-containing regimens for hepatitis C virus in patients with decompensated cirrhosis: a real-world study

**DOI:** 10.1186/s13027-017-0158-1

**Published:** 2017-09-13

**Authors:** Fanpu Ji, Wenjun Wang, Shuangsuo Dang, Shengbang Wang, Burong Li, Dan Bai, Wenxue Zhao, Hong Deng, Changyin Tian, Zongfang Li

**Affiliations:** 1 Department of Infectious Diseases, Second Affiliated Hospital of Xi’an Jiaotong University, 157 Xi Wu Road, Xi’an, 710004 Shaanxi Province People’s Republic of China; 2 Shaanxi Provincial Clinical Research Center for Hepatic & Splenic Diseases, Second Affiliated Hospital of Xi’an Jiaotong University, Xi’an, China; 3 Department of Clinical Laboratory, Second Affiliated Hospital of Xi’an Jiaotong University, Xi’an, China; 4 Department of Biochemistry and Molecular Biology, Medical College of Xi’an Jiaotong University, Xi’an, China; 5 National & Local Joint Engineering Research Center of Biodiagnosis and Biotherapy, Second Affiliated Hospital of Xi’an Jiaotong University, Xi’an, China

**Keywords:** Hepatitis C virus, Sofosbuvir, Child-Pugh score, Decompensated cirrhosis, Renal dysfunction, Anemia

## Abstract

**Background:**

Direct-acting antivirals have been used for decompensated cirrhotic patients with hepatitis C virus (HCV) infection. However, the benefits in Chinese patients with decompensated cirrhosis are unclear.

**Methods:**

Thirty patients with HCV infection and decompensated cirrhosis were administered sofosbuvir-containing regimens at our hospital between April and December 2015. The efficacy and safety of the treatments was determined by sustained virological response at week 12 (SVR 12), change of liver function and adverse events.

**Results:**

The cohort included 13 treatment-experienced and 17 treatment-naïve patients. A total of 27 patients (90%) achieved SVR 12. No baseline characteristics (sex, age, treatment-experience, genotype, viral load, liver function or splenectomy) was association with achievement of SVR 12. Patients achieved SVR 12 had significantly improved liver function by post-treatment week 12 (*P* < 0.05). Of the 30 patients, six developed anemia, one developed hepatic decompensation, two developed impaired renal function and one developed a severe upper respiratory tract infection during the treatment. There was no death or HCC development during 12 months of follow-up off-therapy. Two patients (7.4%) with SVR 12 experienced new decompensated episodes during the follow-up.

**Conclusion:**

Sofosbuvir-containing regimens are effective in Chinese HCV patients with decompensated cirrhosis, regardless of baseline characteristics, as demonstrated by a high rate of SVR 12, as well as improvement in liver function. Although antiviral therapy is generally well tolerated, a vigilant monitoring of anemia and renal function should be mandatory.

## Background

Hepatitis C virus (HCV) infection is a global health problem with an estimated disease burden affecting 130–170 million people. China, with a prevalence of 0.6–2.0%, has the most people (more than 10 million) with chronic HCV infection worldwide [1, 2]. Chronic hepatitis C (CHC) represents a life-threatening condition, especially if left untreated, as it is leads to development of cirrhosis, a liver disease that is accompanied by high risk of progression to hepatic decompensation and hepatocellular carcinoma (HCC) [1–3]. Despite advances in screening strategies and effective antiviral therapy, the number of patients with HCV-related decompensated cirrhosis is projected to rise over the next decade or more [2, 4, 5].

HCV eradication in compensated and decompensated cirrhotic patients after interferon (IFN)-based antiviral therapy is associated with improvement of liver metabolic activity, prevention of HCV recurrence after transplantation, and removal of some patients from the waiting list for liver transplant, as well as reduced risk of HCC development [6–12]. Unfortunately, IFN-based antiviral therapy for cirrhotic patients with advanced disease is also associated with poor tolerability, serious infections and increased risk of death [10–13]. The advent of direct-acting antiviral agents (DAAs) promises to overcome these disadvantages in safety while increasing efficacy.

Sofosbuvir is a nucleotide analogue inhibitor of the HCV-encoded NS5B polymerase, and has been approved for treatment of genotypes 1–4 [14]. Sofosbuvir in combination with other DAAs (such as ledipasvir, daclatasvir, velpatasvir, simeprevir) with or without ribavirin has been reported to attain a sustained virologic response (SVR) rate of over 90% when administered as a 12- to 24-week course, while being well-tolerated in cirrhotic patients with advanced disease as well as in liver transplant patients through both clinical trials and real-world evidence [15–24]. Moreover, the sofosbuvir-based therapy also has been shown to improve liver function and halt liver disease progression in cirrhotic patients with advanced disease [15, 19, 20]. Despite of the impact of DAAs treatments on the risk of HCC occurrence in patients without HCC or on the risk of tumor recurrence after curative treatment of HCC remains controversial [25–29].

To date, few report of DAAs treatment of Chinese patients with HCV infection and decompensated cirrhosis exists in available literature without the data of follow-up off-therapy [30, 31]. Therefore, the objective of present study was to share our real-world experience of effectiveness and safety of sofosbuvir-containing regimens for HCV in patients with decompensated cirrhosis in a tertiary hospital in northwest China.

## Methods

### Study design and patients

This study complied with the Declaration of Helsinki. The institutional review board of the Second Affiliated Hospital of Xi’an Jiaotong University waived the requirement for approval of this retrospective study. Patients were offered study enrollment upon meeting the following inclusion criteria: positive anti-HCV and HCV RNA at last 3 months; on admission, Child-Pugh score ≥ 7 or ≤6 points and at least one pre-admission event signifying hepatic decompensation, including ascites, variceal bleeding, spontaneous bacterial peritonitis (SBP) or hepatic encephalopathy. Patients with HCC, chronic renal failure, unstable cardiovascular disease, severe chronic obstructive lung disease, co-infection with human immunodeficiency virus, or Child-Pugh class C after the decompensated event who had been treated with appropriate therapy were denied study enrollment. Among those patients who met the criteria for study enrollment, only those who were willing to accept and adhere to the prescribed drug and regimen were included in the final study population.

**Fig. 1:**
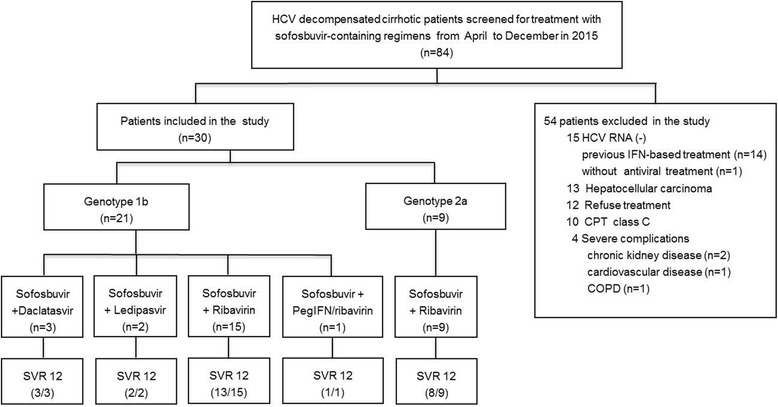
Antiviral therapy regimens and virologic response in patients with decompensated cirrhosis. COPD, chronic obstructive lung disease

In total 84 HCV related decompensated cirrhotic patients were screened for treatment in our hospital from April to December 2015. Fifteen of them were excluded for negative HCV RNA (14 of 15 patients with successful HCV treatment by IFN-based regimens), 13 were excluded for HCC, 12 for refusing to be treated with sofosbuvir-containing regimens, ten for Child-Pugh class C and four for severe complications including chronic kidney disease (*n* = 2), cardiovascular disease (*n* = 1) and severe chronic obstructive lung disease (*n* = 1). The remaining 30 patients were enrolled into the study (Fig. 1) included 13 treatment-experienced patients [8, 11, 32, 33] and 17 treatment-naïve patients.

### Treatment schedules

Antiviral treatment started 1- to 2-weeks after the decompensated event had been treated with appropriate therapy such as antibiotic treatment for SBP, diuretics for ascites and edema, etc. Prior to initiation of the sofosbuvir-containing therapeutic regimens, each patient underwent electrocardiogram, endoscopy, liver computed tomography (CT)/B ultrasound, α-fetoprotein and virus genotyping (Da An Gene Co. Ltd. of Sun Yat-Sen University, China). Twenty-one (70%) of the patients were infected with HCV genotype 1b. Among these patients, two were treated with sofosbuvir (400 mg/d) + ledipasvir (90 mg/d) for 16 weeks, three were treated with sofosbuvir (400 mg/d) + daclatasvir (60 mg/d) for 12–16 weeks, 15 were treated with sofosbuvir (400 mg/d) + ribavirin (900–1200 mg/d) for 24 weeks, and one was treated with triple-combination therapy of sofosbuvir (400 mg/d) + pegylated interferon alpha-2b (Peg-IFN α-2b) (80 μg/wk) + ribavirin (1000 mg/d) for 12 weeks. All of the nine patients with HCV genotype 2a infection were treated with sofosbuvir (400 mg/d) + ribavirin (900 mg/d) for 16–24 weeks. For patient showed decrease in hemoglobin to 9.0–10.0 g/dL required closely monitored, to 8.0–9.0 g/dL required ribavirin dose reductions, to less than 8.0 g/dL required ribavirin discontinuations and received erythropoietin treatment, which was referred to our previous studies of IFN-based treatment for this population [8, 11, 33]. The treatment regimens and effectiveness were presented in Fig. 1.

### Treatment monitoring and follow-up

Patients receiving therapy were reviewed at treatment weeks 2, 4 and 12 and/or end of treatment, and at post-treatment weeks 4 and 12, and then once every 3 months subsequently. Partial patients underwent HCV RNA testing at days 3, and 7 of treatment. The blood cell count, HCV RNA and serum markers including total bilirubin (TBIL), alanine aminotransferase (ALT), albumin (ALB), urea nitrogen and serum creatinine, prothrombin activity (PTA) were checked in each review routinely. All patients received liver B ultrasound and α-fetoprotein test every 3 months after DAAs treatment for HCC screening. And patient also received liver computed tomography with or without contrast enhancement scan when it is necessary to rule out HCC development. HCV RNA was measured using the 7300 real-time PCR system (Applied Biosystems Inc., USA) and reagents from the Da An Gene Co. Ltd. of Sun Yat-Sen University, China. However, the Cobas *Taq*Man quantitative detection kit, with a detection limit <25 IU/mL, was used to measure HCV RNA at end-of-treatment and post-treatment week 12. Serious adverse events (SAEs), decompensation events, severe infections, HCC development and death were recorded. Other common adverse events (AEs), such as palpitation, dizziness, nausea, fatigue etc., were also recorded.

### Outcome measures

The primary end point of our study was sustained virological response at 12 weeks post-treatment (SVR 12), which was defined as undetectable HCV RNA measured with a detection limit of quantification <25 IU/mL. The secondary end points included undetectable HCV RNA at treatment week 1, 2 or 4, change in liver function (serum TBIL, ALT, ALB, PTA and Child-Pugh score) at post-treatment week 12, AEs including liver decompensation and HCC development during the treatment and follow-up period. The length of the follow-up period was calculated from the end of the antiviral treatment to the last follow-up visit.

### Statistical analysis

Data are represented as the mean and standard deviation or as absolute and relative frequencies. Statistical comparisons were carried out using the chi-square test or Fisher’s exact test for baseline characteristics, and the two-sided paired *t*-test for the change in liver function at baseline and post-treatment week 12. The following baseline characteristics were examined in relation to SVR 12: patient age > 60 years, sex, treatment-experienced, HCV genotype, baseline viral load ≥1.0 × 10^6^ IU/mL, Child-Pugh score ≥ 7, and splenectomy. Analyses were carried out using SPSS statistical software, version 17.0 (IBM, Chicago, IL, USA). A *p*-value of <0.05 was set as the threshold for statistical significance.

## Results

### Patient demographics and clinical characteristics

The 30-patient cohort consisted of 8 males and 22 females, with a mean age of 58.57 ± 8.06 years-old (range: 42–73 years-old). Among the 30 patients, 13 (43.3%) had previously been administered an IFN-based regimen and showed treatment failure including relapse and non-virological response; the remaining 17 patients were treatment-naïve. The collective characteristics are presented in Table 1.

**Table 1:** Patient demographics and clinical characteristics

Characteristic	Treatment-naïve patients (*n* = 17)	Treatment-experienced patients (*n* = 13)	All patients, (*n* = 30)
Sex, n (%)			
Male Female	4 (23.5) 13 (76.5)	4 (30.8) 9 (69.2)	8 (26.7) 22 (73.3)
Age, n (%)			
≥ 60 years-old < 60 years-old	7 (41.2) 10 (58.8)	7 (53.8) 6 (46.2)	14 (46.7) 16 (53.3)
Genotype^a^, n (%)			
1b 2a	9 (52.3) 8 (47.7)	12 (92.3) 1 (7.7)	21 (70.0) 9 (30.0)
HCV RNA, n (%)			
≥ 1.0 × 10^6^ IU/mL < 1.0 × 10^6^ IU/mL	7 (41.2) 10 (58.8)	9 (69.2) 4 (30.8)	16 (53.3) 14 (46.7)
Child-Pugh score^a^, n (%)			
≥ 7 < 7	7 (41.2) 10 (58.8)	2 (15.4) 11 (84.6)	9 (30.0) 21 (70.0)
Splenectomy, n (%)			
Yes No	6 (35.3) 11 (64.7)	3 (23.1) 10 (76.9)	9 (30.0) 21 (70.0)

^a^Compared with the treatment-naïve patients, a lower proportion of treatment- experienced patients is genotype 2 and Child-Pugh score ≥ 7; *p* < 0.05

### Virological outcome

SVR 12 was achieved in 27/30 (90%) for this population of HCV decompensated cirrhotic patients. For the various treatment regimens administered (Fig. 1), two of the patients infected with genotype 1b who received the sofosbuvir + ribavirin therapy experienced relapse at weeks 4 and 12 after end-of-treatment respectively, and one of them was treatment-naïve. Another treatment-naïve patient infected with genotype 2a who received sofosbuvir + ribavirin for 16 weeks experienced HCV recurrence at post-treatment week 12.

**Fig. 2:**
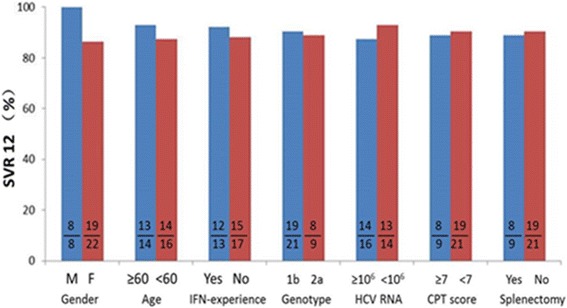
SVR 12 by baseline factor

No baseline factors, including sex (SVR in males, 100% vs. in females, 86.3%), age (SVR in ≥60 years-old, 92.9% vs. <60 years-old, 87.5%), previous IFN-based therapy experience (SVR in treatment-experienced, 92.3% vs. treatment-naïve, 88.2%), virus genotype (SVR in 1b–infected, 90.5% vs. 2a–infected, 88.9%), baseline viral load (SVR in ≥10^6^ IU/mL, 87.5% vs. <10^6^ IU/mL, 92.9%), Child-Pugh score (SVR in ≥7, 88.9% vs. <7, 90.5%) and previous splenectomy treatment (SVR in splenectomy, 88.9% vs. no splenectomy, 90.5%) was associated with the achievement of SVR 12 (all *p* > 0.05) (Fig. 2).

Serum HCV RNA was detected in 12 patients 3 days after receiving antiviral treatment with sofosbuvir-containing regimens. There was an average reduction in HCV RNA viral load by 3.34 log10 (baseline, 6.06 ± 0.62 vs. treatment day 3, 2.72 ± 1.33). The rates of undetectable HCV during treatment were 54.8% (7/13) at week 1, 83.3% (15/18) at week 2, 100% (30/30) at week 4 and 100% (30/30) at end-of-treatment.

### Effects of sofosbuvir-containing regimens on liver function and safety

The sofosbuvir-containing treatment led to significant improvements in liver function (baseline vs. post-treatment week 12; Table 2). Totally, 14 patients (46.7%) experienced at least one AEs, four patients (13.3%) experienced SAEs. Of these SAEs, two patients experienced increase in serum urea nitrogen and/or serum creatinine to levels above the upper limit of normal. Another one patient developed severe anemia (5.6 g/dL in hemoglobin) accompanied by liver decompensation, massive ascites, and lower extremity edema; the complications were severe enough to require hospitalization and a 3-week discontinuation of the ribavirin therapy. The remains patient developed a severe upper respiratory tract infection, which was resolved with cefatriaxone treatment, administered for 5 days.

**Table 2:** Liver function markers pre- and post-treatment week 12 (*n* = 30)

	TBIL, in μmol/L	ALT, in IU/L	ALB, in g/L	PTA, in %	Child-Pugh score
Baseline	23.48 ± 12.54	50.17 ± 32.93	39.52 ± 5.73	76.17 ± 16.80	6.30 ± 1.60
Post-treatment week 12	18.98 ± 9.24	25.70 ± 10.67	42.96 ± 6.17	79.85 ± 14.62	5.87 ± 1.14
*t-*value	3.371	3.973	4.214	2.544	2.642
*p*-value	0.002	0.000	0.000	0.017	0.013

*ALB* albumin, *ALT* alanine aminotransferase, *PTA* prothrombin activity, *TBIL* total bilirubin

Anemia (20%) was the most common AEs, all of the six patients experienced a more than 2.0 g/dL decrease in hemoglobin, who received a treatment regimen containing ribavirin (*n* = 25). Among them, four and two patients showed decrease in hemoglobin to less than 10.0 g/dL and 8.0 g/dL, respectively. The former responded to a reduction in the ribavirin dosage, while the other two required to interrupted ribavirin and received erythropoietin treatment. Of the 25 patients received ribavirin-containing regimens treatment, the hemoglobin was significantly reduced at the end-of-treatment, compare to those at baseline (11.5 ± 1.08 vs. 10.7 ± 0.96 g/dL, *P* = 0.013). Despite the hemoglobin re-elevated to a level of similar to baseline at 12 weeks post-treatment (11.5 ± 1.08 vs. 11.6 ± 0.99 g/dL, *P* = 0.546). There were no different of hemoglobin at baseline, end-of-treatment and 12 week post-treatment for five patients treated with ribavirin-free regimens. Other AEs included nausea, fatigue, palpitation, pruritus/rash, dizziness/headache, shortness of breath, and sleep disorders (Table 3).

**Table 3:** Summary of adverse event during the treatment period (*n* = 30)

Patients with adverse event	Number (%)
Any adverse event	14(46.7)
Serious adverse event	4(13.3)
Renal dysfunction	2(6.7)
Hepatic decompensation	1(3.3)
Upper respiratory tract infection	1(3.3)
Discontinuation due to adverse event	3(10)
Death	0(0)
Common adverse event	
Anemia	6(20)
Nausea	3(10)
Fatigue	2(6.7)
Palpitation	2(6.7)
Pruritus/rash	2(6.7)
Dizziness/headache	2(6.7)
Shortness of breath	1(3.3)
Sleep disorders	1(3.3)

### Outcome of follow-up off-therapy

There was no death or HCC development during antiviral therapy and the median 12 (range 8–15) months of follow-up off-therapy. And none of the patients need for liver transplant, although two of the 27 patients with SVR 12 experienced new decompensated episodes of encephalopathy and ascites in 4 and 9 month after end-of-treatment, respectively.

## Discussion

Overall estimates indicate that up to 15% of patients with chronic HCV infection progress to cirrhosis within 20 years. Liver cirrhosis progression to the decompensated stage has an annual incidence of about 3–4% [1, 2]. Once decompensated HCV-related cirrhosis is established, patients have a poor prognosis, and are characterized by a very high frequency of readmission, development of decompensation different from the initial one, a generally low quality of life, and high risk of mortality [1, 2, 34, 35]. Thus, a highly safe and effective regimen for all genotypes of HCV infection in patients with decompensated liver disease would address a significant unmet medical need.

Studies have shown that more than 90% patients achieve SVR 12 after DAAs treatment with or without ribavirin, and the treatment led to early improvements in hepatic function [15, 16, 18–20]. Therefore, the European Association for Study of Liver (EASL) does not recommend IFN-based regimens for patients with decompensated cirrhosis; instead, patients with genotype 2 infections are recommend to be treated with sofosbuvir + ribavirin for 16–20 weeks, and patients with genotype 1 infections are recommend to be treated with sofosbuvir + ledipasvir or sofosbuvir + daclatasvir with or without ribavirin for 12 or 24 weeks [36]. The treatment combination of sofosbuvir + velpatasvir has also been shown to have excellent efficacy and safety profiles in patients with decompensated cirrhosis [15]. In general, however, studies of DAAs in Chinese populations are lacking, due to the slow approval process and high cost of DAAs drugs. And our findings presented herein provide the clinical experience of sofosbuvir-containing regimens for Chinese patients with hepatitis C and decompensated cirrhosis.

Our results show that the sofosbuvir-containing treatment regimens are well tolerated in this patient population, and provide a very good efficacy, regardless of baseline characteristics. All of the 27 patients in our study who achieved SVR 12 continued to show undetectable levels of HCV RNA up to post-treatment week 24. These results are in agreement with the recently reported multi-center clinical research study from China and Korea that showed that patients with genotype 1b infection who are ineligible/intolerant to IFN therapies respond well to daclatasvir + asunaprevir combination therapy [37]. In our study, patients who achieved SVR 12 showed significant improvements in liver function, including TBIL, ALT, ALB, PTA and Child-Pugh score post-treatment week 12, which was consistent with the previous studies [15, 16, 18–20]. However, the longer term impact of DAAs treatment in patients with decompensated cirrhosis remains unknown. Sustained virologic response to IFN-based antiviral therapy decreases the incidence of HCC and the risk of hepatic decompensation in patients with HCV cirrhosis [6–11]. SVR after DAAs treatment reduces the incidence of HCC in patients with CHC and compensated cirrhosis had been reported recently [26]. For patients with decompensated cirrhosis, Cheung et al. suggested antiviral therapy led to prolonged improvement in liver function without evidence of an increased risk of HCC development after 15 months follow-up off-therapy [19]. However, other studies showed unexpected high incidence of HCC in cirrhotic patients with SVR following IFN-free DAAs treatment [25, 27, 28]. None of our patients occurred HCC or death, and only 7.4% of patients with SVR 12 experienced new decompensated episodes during the median 12 (range 8–15) months of follow-up off-therapy. Thus, patient with decompensated cirrhosis can benefit from antiviral therapy with DAAs.

Although ribavirin treatment was not associated with hepatic decompensation, 30.4% and 5.4% patients with compensated and decompensated cirrhosis need ribavirin dose reductions and discontinuations respectively [38]. Anemia was the most common AEs, and all of them presented in patients with ribavirin-containing regimen in our study. Thus, it appears that the ribavirin component may be responsible for the side effect of anemia. However, all of the six patients with ribavirin dose reductions and/or discontinuations achieved SVR 12, which add further evidence of ribavirin dose adjustment in cirrhotic patients with advanced disease should not reduce efficacy of antiviral treatment by Saxena et al. [38].

Two additional patients experienced renal adverse reaction, as evidenced by increased serum urea nitrogen/creatinine and decreased glomerular filtration rate (GFR). Both of them responded to a 2-week discontinuation of antiviral treatment, along with management using benazepril and prostaglandin E1. Re-initiation of the sofosbuvir + ribavirin treatment, after the patients showed improvement of renal dysfunction, were well tolerated. Ultimately, both of the patients completed the antiviral treatment and achieved SVR 12. Sofosbuvir is primarily metabolized by the kidney. Studies have shown that patients with HCV infection and decompensated cirrhosis and who underwent liver transplantation can experience reduced GFR or even acute renal failure during pre- and post-transplantation therapy with a sofosbuvir-based regimens [15, 18, 38]. However, studies also had shown that sofosbuvir-based regimen using dosage of 400 mg is effective and safe in patients with end-stage renal disease undergoing hemodialysis or who have GFR < 30 mL/min [39, 40].

Considering the potential risk of hepatorenal syndrome or acute kidney injury in patients with end-stage liver disease, and both sofosbuvir and ribavirin are mainly through kidney metabolism, patients with hepatitis C infection and decompensated cirrhosis who are administered a sofosbuvir-containing regimen with or without ribavirin require close monitoring of renal function. If renal damage occur or aggravate, treatments should be given to improve renal function, and short interruption or dose reductions of antiviral treatment may be necessary [38], as was evidenced in cases in our study.

Limits of this retrospective study include heterogeneity of antiviral treatments, a relatively small number of cases, limited length of follow-up and possible selection bias. In particular, due to the urgency of treatment, the accessibility of drugs during the study period, as well as the rapid movement in the field of anti-HCV treatment and the dramatic improvement in efficacy and safety of DAAs, not all patients received the antiviral therapy program recommended by current guidelines [36, 41, 42], such as 15 GT 1-infected patients received sofosbuvir + ribavirin treatment for 24 weeks. Again, due to the nature of retrospective study, there were different sofosbuvir-containing regimens with or without ribavirin, which potential preclude useful comparison in the statistical analysis. Furthermore, we enrolled only patients with mild to moderate liver decompensation, so our results cannot be generalized to patients with more severe liver disease. Finally, these results also cannot be extrapolated widely to all patients with mild to moderate liver decompensation. Despite these limitations, several conclusions can be drawn based upon careful consideration of the available data.

## Conclusions

Sofosbuvir-containing regimens produced high rates of SVR 12 in decompensated cirrhotic patients, regardless of baseline characteristics. Antiviral treatment was associated with improvement in liver function, and was generally well tolerated in this Chinese population. However, a vigilant monitoring of anemia and renal dysfunction should be mandatory. Results from currently ongoing large and well-designed long-term study (ClinicalTrials.gov number, NCT01457755) [15] is awaited to verify the benefit and safety of sofosbuvir-containing regimens for decompensated cirrhotic patients with HCV infection including the risk of HCC development and long-term survival.

## Abbreviations

ALB: Albumin
ALT: Alanine aminotransferase
CHC: Chronic hepatitis C
DAAs: Direct-acting antiviral agents
GFR: Glomerular filtration rate
HCC: Hepatocellular carcinoma
HCV: Hepatitis C virus
PTA: Prothrombin activity
SBP: Spontaneous bacterial peritonitis
SVR 12: Sustained virological response at week 12
TBIL: Total bilirubin
